# Trait-dependent resemblance of the flowering phenology and floral morphology of the allopolyploid *Cardamine flexuosa* to those of the parental diploids in natural habitats

**DOI:** 10.1007/s10265-019-01164-0

**Published:** 2020-01-10

**Authors:** Reiko Akiyama, Stefan Milosavljevic, Matthias Leutenegger, Rie Shimizu-Inatsugi

**Affiliations:** Department of Evolutionary Biology and Environmental Studies, University of Zurich, Winterthurerstrase 190, 8057 Zurich, Switzerland

**Keywords:** *Cardamine*, Floral morphology, Flowering phenology, Polyploid, Reproductive traits, Trait variation

## Abstract

Allopolyploids possess complete sets of genomes derived from different parental species and exhibit a range of variation in various traits. Reproductive traits may play a key role in the reproductive isolation between allopolyploids and their parental species, thus affecting the thriving of allopolyploids. However, empirical data, especially in natural habitats, comparing reproductive trait variation between allopolyploids and their parental species remain rare. Here, we documented the flowering phenology and floral morphology of the allopolyploid wild plant *Cardamine flexuosa* and its diploid parents *C. amara* and *C. hirsuta* in their native range in Switzerland. The flowering of *C. flexuosa* started at an intermediate time compared with those of the parents and the flowering period of *C. flexuosa* overlapped with those of the parents. *Cardamine flexuosa* resembled *C. hirsuta* in the size of flowers and petals and the length/width ratio of petals, while it resembled *C. amara* in the length/width ratio of flowers. These results provide empirical evidence of the trait-dependent variation of allopolyploid phenotypes in natural habitats at the local scale. They also suggest that the variation in some reproductive traits in *C. flexuosa* is associated with self-fertilization. Therefore, it is helpful to consider the mating system in furthering the understanding of the processes that may have shaped trait variation in polyploids in nature.

**Electronic supplementary material:**

The online version of this article (10.1007/s10265-019-01164-0) contains supplementary material, which is available to authorized users.

## Introduction

Allopolyploids (organisms possessing complete sets of genomes derived from different parental species) exhibit large variation in traits (Ramsey and Ramsey 2014). In controlled conditions, the phenotypes of synthetic neo-allopolyploids and natural allopolyploids were either similar, intermediate, or transgressive compared with those of the parental species (Abbott and Lowe 2004; Alexander-Webber et al. 2016; Davis 1943; Schranz and Osborn 2004). Such findings do not necessarily translate to natural allopolyploids in natural habitats, because their trait variation is likely influenced by environmental conditions and potentially by adaptive evolution and genetic drift in the local habitats. The few studies that have focused on individuals in natural habitats report that the phenotypes of allopolyploids are intermediate or transgressive (Allen 2001; Vallejo-Marín 2012). However, trait variation in allopolyploids and their parental species in natural habitats remains largely unexplored (Ramsey and Ramsey 2014), partly because of the difficulty to identify the diploid progenitors of an allopolyploid (Buggs et al. 2014).

Trait variation in allopolyploids may be shaped by inheritance, polyploidization, and natural selection. The influence of inheritance and polyploidization should be prominent in allopolyploids shortly after emergence, while natural selection should affect trait variation over a longer time. Strong inheritance is observed in *Senecio cambliensis*, which is estimated to have emerged within the last 200 years (Brennan and Hiscock 2010). Allopolyploidization may confer large organ size and delayed flowering (Garbutt and Bazzaz 1983; Stebbins 1971; te Beest et al. 2012), although such effects may diminish after several generations (Gaeta et al. 2007). In allopolyploids with an old origin, on the other hand, trait variation is likely shaped by natural selection (Ramsey and Ramsey 2014), although it is impossible to determine the relative contributions of inheritance, allopolyploidization, and natural selection to the current trait variation in allopolyploids.

Variation in reproductive traits in polyploids may be shaped by their mating system. Many polyploids are self-fertilizing (Barringer 2007), which can contribute to the persistence of polyploids through reproductive assurance (Mable 2004; Miller and Venable 2000) and through reproductive isolation from the parental species because the success of reproduction of polyploids is independent from parents (Soltis et al. 2010 and references therein). Compared with outcrossing species, it is less important for self-fertilizing species to have large floral organs or synchronized flowering, which can be advantageous for attracting pollinators (e.g., Conner and Rush 1993; Elzinga et al. 2007; Forsyth 2003; Parachnowitsch and Kessler 2010). In fact, self-fertilizing species typically exhibit the selfing syndrome, in which the floral organs are smaller (Foxe et al. 2009; Shimizu and Tsuchimatsu 2015; Sicard and Lenhard 2011; Tedder et al. 2015a) compared with those of their outcrossing counterparts. Self-fertilizing species also exhibit an earlier onset of flowering on calendar days in the same season (Martin and Willis 2007; Mazer et al. 2004). Small floral organs and early onset of flowering is expected from self-fertilizing allopolyploids with old origin. Given the long amount of time since allopolyploidization, selfing-syndrome should have manifested stronger, whereas there may no longer be a large effect of allopolyploidization, which results in large organ size and delayed flowering. To examine whether self-fertilizing allopolyploids exhibit variation in reproductive traits in accordance with the selfing syndrome, it is essential to gather empirical data on allopolyploids and parental species with a known mating system and estimated time of allopolyploidization.

The genus *Cardamine* provides a convenient system for evolutionary ecological studies of allopolyploids. *Cardamine* is one of the largest genera in Brassicaceae and has experienced recurrent polyploid speciation, with more than half of the species estimated to be polyploid (Carlsen et al. 2009; Howard 1948; Hussein 1948; Kučera et al. 2005; Lihová and Marhold 2006; Lihová et al. 2006a, b; Mandáková et al. 2014; Zozomová-Lihová et al. 2014). The genus has been the subject of study from several aspects, such as genetics (Hay et al. 2014), genomics (Gan et al. 2016), ecological transcriptomics (Shimizu-Inatsugi et al. 2017), and morphology (e.g., Lihová et al. 2006b, 2007; Marhold 1992, 1998). While multiple species have been surveyed in *Cardamine*, there is little documentation of trait variation specifically in allopolyploids compared with their diploid parents in natural habitats.

We conducted a local-scale field observation to quantify the variation in floral morphology and flowering phenology of a *Cardamine* allopolyploid and its parents in their native habitats in Switzerland. Populations of the three species included in the study occurred sympatrically in the same climatic region within a distance of 12 km. *Cardamine flexuosa* is an allotetraploid originating from the diploids *C. amara* and *C. hirsuta*, presumably around 10^5^–10^6^ years ago (Mandáková et al. 2014). While *C. amara* is predominantly outcrossing (Tedder et al. 2015b) and is pollinated by insects such as beetles and flies (Kentaro K. Shimizu, Akiko Yasumoto, and Reiko Akiyama, personal observation), *C. hirsuta* and *C. flexuosa* are self-fertilizing (Hay et al. 2014; Reiko Akiyama, personal observation). The three species have different preferences to drought and submergence (Shimizu-Inatsugi et al. 2017). This seemingly reflects the environmental conditions of each species’ habitat, with the diploid parents at either extreme and the allopolyploid in an intermediate and wide range (Akiyama et al. 2019). In this study, we addressed the following questions. (1) How do the reproductive traits of the allopolyploid compare with those of the parents in natural habitats? (2) Is the relationship in trait variation between the allopolyploid and its parents consistent for all reproductive traits, or does it vary among traits? If the latter is true, how does it vary?

## Materials and methods

### Study species

*Cardamine amara* L. (large bittercress) is a perennial herb that is widespread across central Europe and has served recurrently as a parent of polyploid species (Grime et al. 2007; Lihová et al. 2000, 2006b; Mandáková et al. 2014; Marhold 1998; Zozomová-Lihová et al. 2014). In Switzerland, the populations of *C. amara* in lowland areas (< 1,000 m) are reported to consist of diploids (2*n* = 2*x* = 16), and autotetraploid populations of the subsp. *austriaca* have been observed at higher altitudes in the inner Alpine valleys (Marhold 1995, 1999; Marhold et al. 2002). *Cardamine amara* propagates clonally and sexually (Tedder et al. 2015b). In addition, in many populations in Switzerland, the coexistence of female-sterile individuals with short pistils and hermaphrodite individuals with normal-length pistils has been reported (Tedder et al. 2015b), which is a rare reproductive system called androdioecy. *Cardamine amara* typically grows 10–60 cm in height and does not form a compact rosette (Lauber et al. 2012). The color of anthers is red for some individuals, but occurrences of yellow anthers have been observed (Reiko Akiyama, personal observation). The petal is white in most cases, though it can be purplish at basal parts in some cases (Reiko Akiyama, personal observation; Lauber et al. 2012).

*Cardamine hirsuta* L. (hairy bittercress) is a diploid (2*n* = 2*x* = 16) annual herb native to Europe (Grime et al. 2007; Lihová et al. 2006b; Marhold 1995; Yatsu et al. 2003). It grows 5–30 cm in height and forms a rosette (Lauber et al. 2012). The species has yellow anthers and white petals (Reiko Akiyama, personal observation).

*Cardamine flexuosa* With. (wavy bittercress) is a tetraploid (2*n* = 4*x* = 32) annual or perennial herb native to Europe (Lihová et al. 2006b; Reiko Akiyama, personal observation) derived from *C. hirsuta* and *C. amara* (Grime et al. 2007; Mandáková et al. 2014). It is distinguished from the formerly named Asian *C. flexuosa*, which now belongs to a distinct octoploid species, *C. occulta* (Lihová et al. 2006b; Marhold et al. 2016). It grows up to 30 cm in height and forms a rosette (Post et al. 2011). The species has yellow anthers and white petals (Reiko Akiyama, personal observation).

### Study sites

The study was conducted in three areas: Irchel (IR, N 47° 23′, E 08° 33′; altitude, 484–505 m), Wehrenbach (WBH, N47° 21′, E 08° 33′; altitude, 422–429 m), and Küsnacht-Tobel (KT, N 47° 19′, E 08° 38′; altitude, 615–664 m) in and around Zurich, Switzerland (Fig. S1a). These areas are located within 4–12 km of each other (Fig. S1a). Each area had two species of the three possible combinations of co-occurring species and all combinations were observed (Fig. S1b–d). For studying flowering phenology, we selected 18 sites (IR1–9, WBH1–3, and KT1–5, 7) in the three areas (Fig. S1b–d) in 2013. WBH1–3 are geographically close, but WBH 3 is separated from WBH 1–2 by a hedge and a paved road. In 2014, flowering phenology was recorded in the same sites as in 2013, with the exception of KT6, which was newly added, and IR9, which was excluded because of the disappearance of the site due to land use. The number of sites studied was 11 for *C. hirsuta*, eight for *C. flexuosa*, and six for *C. amara* in 2013 and 10 for *C. hirsuta*, eight for *C. flexuosa*, and five for *C. amara* in 2014 (Table S1). Table S1 summarizes the composition of the species and the number of individuals of each species per site. For floral morphology, two or three sites per species were subjected to the survey: IR2 and IR8 for *C. hirsuta*; IR2, KT5, and KT6 for *C. flexuosa*; and WBH1 and KT5 for *C. amara* (Fig. S1, Table S1).

When not self-evident, *C. flexuosa* and *C. hirsuta* were identified using morphological keys (Post et al. 2011). We also screened a total of 115 plants in the study sites regarding the ploidy level using flow cytometry (CyFlow^®^ Space, Sysmex Europe GmbH, Norderstedt, Germany). For this analysis, the median of the sample size of each species per site was three. As we found no triploids, the incidence of backcrossing seemed to be low, although gene flow between species cannot be completely excluded (Kolář et al. 2017; Petit et al. 1999).

### Flowering phenology

To compare the onset of flowering in the allopolyploid *C. flexuosa* with that of the parents *C. amara* and *C. hirsuta*, we recorded the flowering status (whether the plant had an open flower(s) on the day of the census) during the season in 2013 and 2014 using different methods. In 2013, prior to the onset of flowering, we marked up to 55 plants per species per site from March to June by randomly selecting plants of a representative size across each site. Because the plants were originally marked for another purpose, the census intervals were irregular (intervals of 1–14 days) and, in most of the censuses, not all marked plants were scored. The total number of plants scored at each census ranged from 13 to 77 for *C. amara*, 37–106 for *C. flexuosa*, and 72–398 for *C. hirsuta* (Fig. S2). In 2014, we marked up to 48 plants per species per site by randomly selecting plants of a representative size across each site on March 4 and recorded the flowering status of these plants weekly from March 5 to June 26 at 6–8-day intervals. When marked plants were lost because of, for example, flooding or removal of the marking by disturbance during the period of study, they were excluded from the analyses. The total number of plants analyzed was 103 for *C. amara*, 136 for *C. flexuosa*, and 195 for *C. hirsuta* (Table S1). In addition, we recorded the flowering status of all plants per site on April 10 (Julian date 100) and May 21 (Julian date 141), to assess the representativeness of the data from the weekly census of a limited number of individuals. In total, we surveyed 4,275 *C. amara* individuals, 816 *C. flexuosa* individuals, and 1,480 *C. hirsuta* individuals on April 10; and 2,920 *C. amara* individuals, 418 *C. flexuosa* individuals, and 188 *C. hirsuta* individuals on May 21.

### Floral morphology

To compare the floral morphology of the study species, we sampled and quantified the size of flowers and petals during the growth season in 2016. The date of survey ranged from April 5 to May 25, according to the flowering phenology of the species. For each site, we haphazardly sampled ~ 20 plants of a representative size for *C. hirsuta* and *C. flexuosa*, and the male and hermaphrodite forms of *C. amara* were distinguished based on pistil length. We collected one to four flowers per plant and photographed them from above at 7 cm (Fig. S3). If possible, we avoided the first and last three flowers and any flowers that seemed abnormal, to capture representative flowers of the plant. Once photographed, the flowers were placed on 0.5% agarose gel to prevent wilting and transported to the laboratory at the University of Zurich, where they were dissected for petal specimens. The specimens were then scanned with a ruler using Epson Perfection^®^ V600 Photo (Epson America Inc.) at 800 dpi (Fig. S3). Using the photographs and scans, the length and width of flowers and petals were measured as the longest axis of the flower seen from above and the direction perpendicular to it, respectively, using ImageJ 1.51a (Rasband 2016). We measured one to four flowers per plant and one representative petal per flower, to calculate the average trait value of the individual. The number of individuals for flower measurement was as follows: flower data, *C. amara* hermaphrodite, *N* = 31; *C. amara* male, *N* = 28; *C. flexuosa*, *N* = 55; *C. hirsuta*, *N* = 38. The number of individuals for petal measurement was as follows: *C. amara* hermaphrodite, *N* = 39; *C. amara* male, *N* = 32; *C. flexuosa*, *N* = 55; *C. hirsuta*, *N* = 40.

### Statistical analysis

All analyses were conducted using R version 3.3.3 (R Core Team 2017). To examine whether flowering phenology differed between species, we conducted two types of tests: the Kolmogorov–Smirnov test and a linear model. A two-sample Kolmogorov–Smirnov test was conducted to compare the distribution of the proportion of the flowering plants between a pair of species each in 2013 and 2014 using the package *stats*, with 0.017 as a threshold *P* value to adjust for multiple tests (0.05 divided by three pairwise tests). In the linear model, the Julian date on which the first flower of the plant opened was regressed upon the species using the package *stats*. Only the data from 2014 were subjected to this analysis. Because of unequal number of individuals in different groups, the data were analyzed with Type III ANOVA using the package *car* (Fox and Weisberg 2011). We conducted a post hoc test with Tukey’s HSD adjustment using the package *multcomp* (Hothorn et al. 2008).

To examine the variation of floral morphology among species and sex, we ran linear models with four groups of species using sex combination (*C. amara* hermaphrodite, *C. amara* male, *C. flexuosa* hermaphrodite, and *C. hirsuta* hermaphrodite) as an explanatory variable and the mean flower length, mean flower width, length/width ratio of a flower, mean petal length, mean petal width, and mean length/width ratio of a petal as response variables. The means were calculated as the average among individuals. In the linear models, the response variables were log transformed prior to the analysis, to meet the assumption of normality. As the data consisted of unequal number of individuals in different groups, we used Type III ANOVA with the package *car* (Fox and Weisberg 2011). When significant difference was detected, post hoc tests were conducted using Tukey’s HSD adjustment with the package *multcomp* (Hothorn et al. 2008).

## Results

### Flowering phenology

**Fig. 1:**
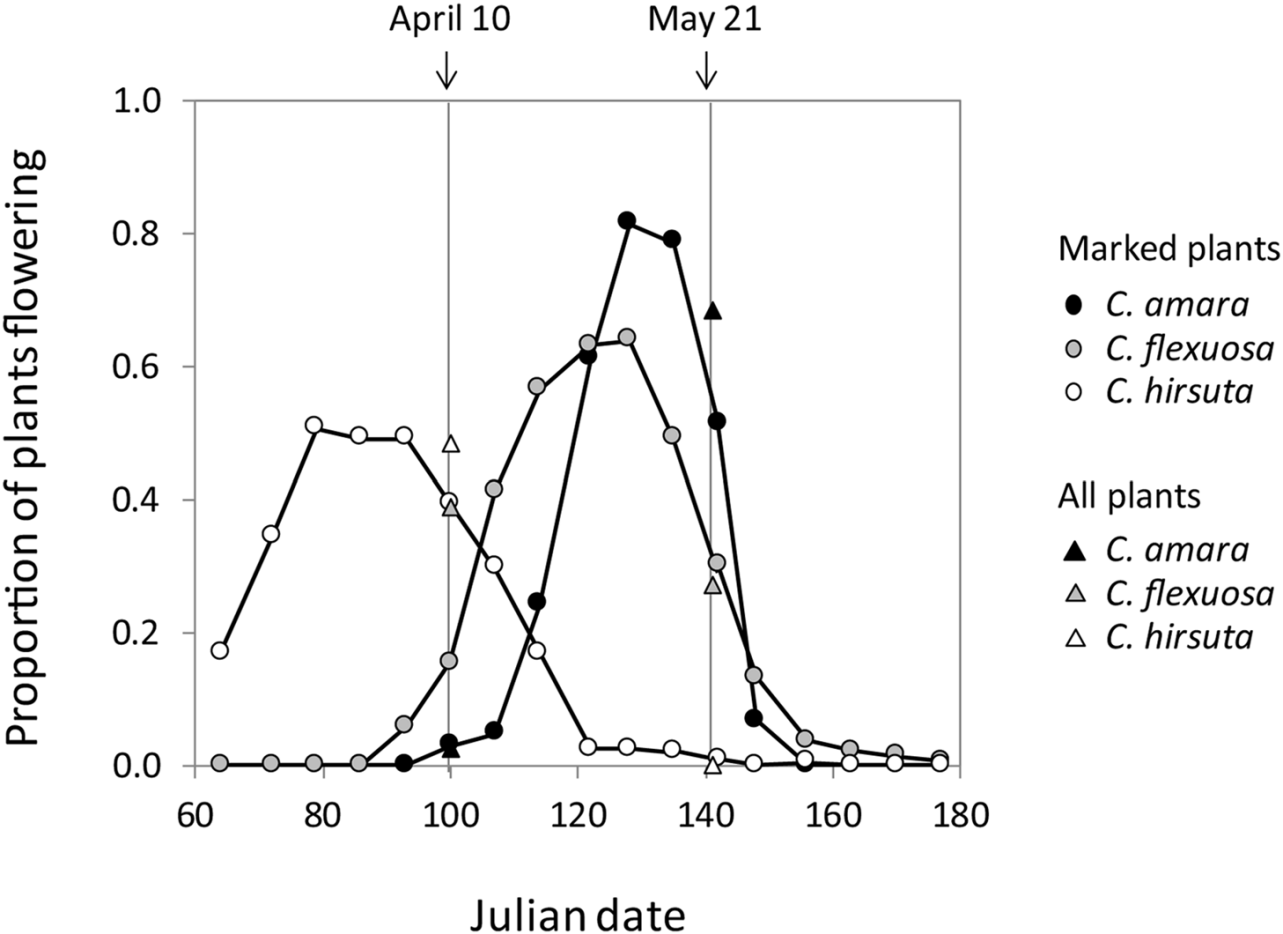
Proportion of flowering individuals of *Cardamine amara*, *C. flexuosa*, and *C. hirsuta* in all study sites in Switzerland in 2014. The circles represent weekly census on marked individuals (*C. amara*, *N* = 113; *C. flexuosa*, *N* = 136; *C. hirsuta*, *N* = 195), while triangles represent census on all individuals at the study sites on April 10 (*C. amara*, *N* = 4,275; *C. flexuosa*, *N* = 812; *C. hirsuta*, *N* = 8,140) and May 21 (*C. amara*, *N* = 2,920; *C. flexuosa*, *N* = 418; *C. hirsuta*, *N* = 188) (Table S1)

In the two seasons, the flowering phenology of *C. flexuosa* was intermediate compared with *C. amara* and *C. hirsuta*. In 2014, the peak of flowering occurred first in *C. hirsuta*, followed by *C. flexuosa* and *C. amara* (Fig. 1). The onset of flowering varied significantly among species (Type III ANOVA, *F*_2, 303_ = 207.75, *P* < 0.0001), with *C. hirsuta* flowering significantly earlier than *C. flexuosa* (Tukey’s HSD test, *P* < 0.0001), which started flowering significantly earlier than *C. amara* (Tukey’s HSD test, *P* < 0.0001). The distribution of the flowered plants did not differ between species (Kolmogorov–Smirnov test, *C. amara* vs *C. flexuosa*: *Z* = 0.33, *P* = 0.27; *C. flexuosa* vs *C. hirsuta*: *Z* = 0.17, *P* = 0.96; and *C. hirsuta* vs *C. amara*: *Z* = 0.28, *P* = 0.49). The census on all plants showed that, on Julian date 100 (April 10), the proportion of plants with open flowers was highest for *C. hirsuta*, followed by *C. flexuosa* and *C. amara*, and that this order was reversed on Julian date 141 (May 21) (Fig. 1). This trend was consistent with that of marked plants on Julian dates 100 and 142 (April 10 and May 22). Thus, the marked plants captured a representative phenology of the study species. Similar to that observed for 2014, in 2013 the peak of flowering was observed first in *C. hirsuta*, followed by *C. flexuosa* and *C. amara* (Fig. S2) and there was no between-species difference in the distribution of the flowered plants (Kolmogorov–Smirnov test, *C. amara* vs *C. flexuosa*: *Z* = 0.41, *P* = 0.36; *C. flexuosa* vs *C. hirsuta*: *Z* = 0.27, *P* = 0.98; and *C. hirsuta* vs *C. amara*: *Z* = 0.55, *P* = 0.26).

In sites that were cohabited by two species, *C. hirsuta* flowered earlier than *C. flexuosa* and *C. flexuosa* flowered earlier than *C. amara* (Fig. S4), indicating that the difference detected using species-level comparisons (Fig. 1) reflected differences between species, rather than between sites, as far as the study sites are concerned.

In *C. amara*, the period of flowering was similar among sites, with a different composition of males and hermaphrodites (Fig. S4): almost all individuals (> 95%) in WBH1 were male, whereas males and hermaphrodites were mixed at a higher ratio in the other populations (Bachmann et al., unpublished).

## Floral morphology

**Fig. 2:**
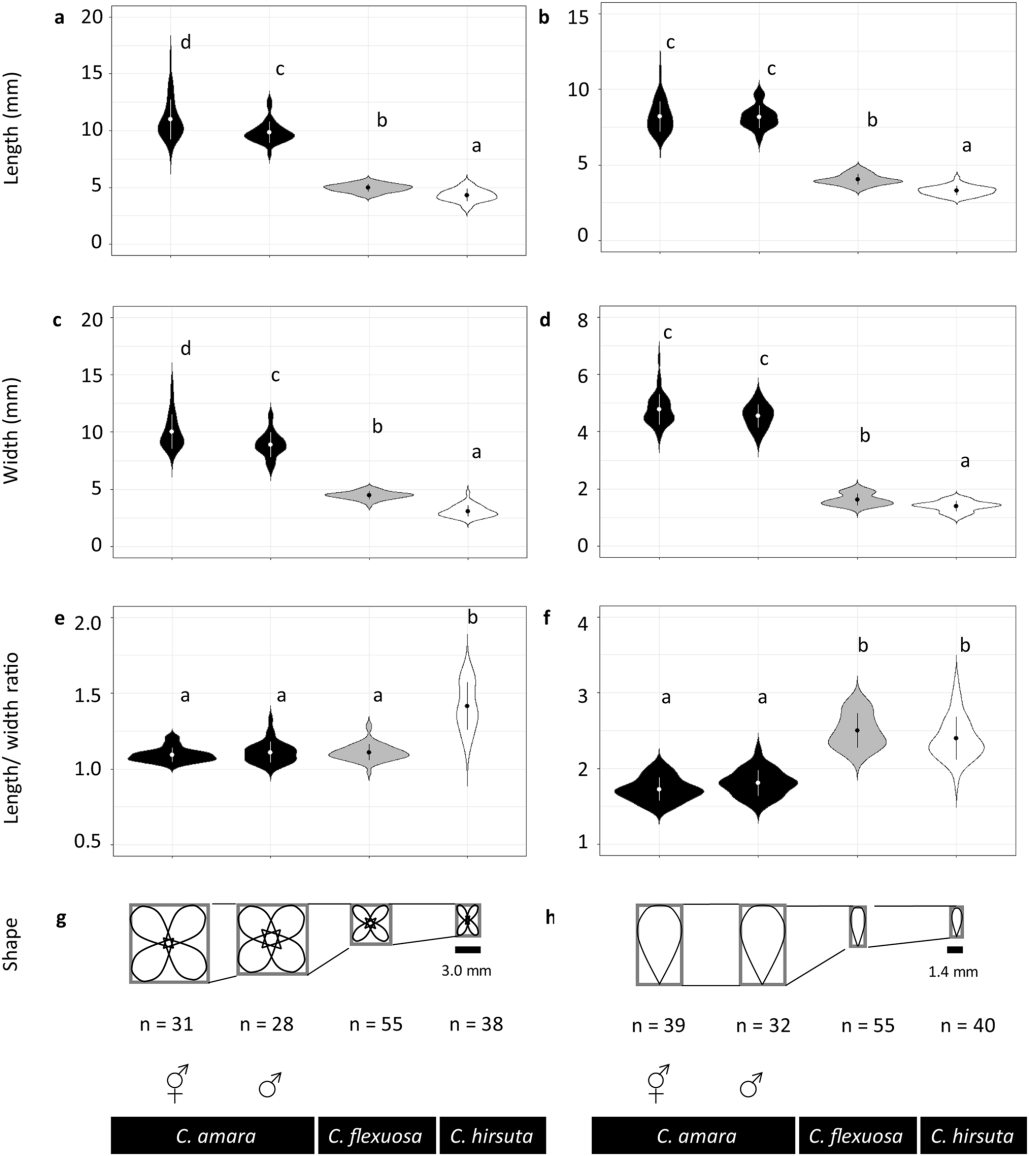
Dimension, length/width ratio of flowers and petals, and schematic drawings of flower and petal morphology of *Cardamine amara* (hermaphrodite and male), *C. flexuosa*, and *C. hirsuta*. **a** Flower length, **b** petal length, **c** flower width, **d** petal width, **e** length/width ratio of the flowers, **f** length/width ratio of the petals, **g** schematic drawing of flower shape based on the mean length and width of the flowers, and **h** schematic drawing of petal shape based on the mean length and width of the petals. For **a**–**f**, the dots and vertical lines within violin plots indicate the mean and SD, and the lowercase letters in each figure indicate statistical differences based on Tukey’s HSD test

The size of flowers and petals varied among species and between sexes (Type III ANOVA, length: *F*_3, 159_ = 706.13, *P* < 0.0001; width: *F*_3, 159_ = 777.68, *P* < 0.0001). The length and width of flowers were largest in the hermaphrodite *C. amara*, followed by the male *C. amara*, *C. flexuosa*, and *C. hirsuta* (Tukey’s HSD tests, Fig. 2a, c). The hermaphrodite and male flowers of *C. amara* were approximately twice as large as the flowers of *C. flexuosa* and *C. hirsuta* (Fig. 2a, c). The shape of flowers also varied among species and sexes (Type III ANOVA, *F*_3, 159_ = 116.12, *P* < 0.0001). The flowers of *C. amara* and *C. flexuosa* exhibited a shape that was close to a square, while the flowers of *C. hirsuta* were more rectangular (Tukey’s HSD tests, Fig. 2e, g).

The petal size varied among species (Type III ANOVA, length: *F*_3, 162_ = 930.48, *P* < 0.0001; width: *F*_3, 162_ = 1204.1, *P* < 0.0001). The length and width of petals were largest in *C. amara*, followed by *C. flexuosa* and *C. hirsuta* (Tukey’s HST tests, Fig. 2b, d). The petal shape of *C. amara* was different from the petal shapes of *C. flexuosa* and *C. hirsuta* (Type III ANOVA, *F*_3, 162_ = 160.03, *P* < 0.0001), as *C. amara* petals were proportionally wider compared with the other two species (Tukey’s HST test, Fig. 2f, h). The hermaphrodites and males of *C. amara* did not differ in petal size (Fig. 2b, d), indicating that the difference in flower size observed between sexes (Fig. 2a, c) can be attributed to the extent of petal opening.

The examination of the sites individually, especially the ones in which *C. flexuosa* cohabited with either of the parents, revealed that the difference, or lack thereof, between species regarding flower and petal morphologies persisted (Figs. S5, S6). These results indicate that the differences detected among species (Fig. 2) reflected differences between species, rather than between sites, at least for the sites included in this study.

## Discussion

The results of the present study provide empirical documentation of trait variation in an allopolyploid and its parents in natural habitats at the local scale. Overall, *C. flexuosa* resembled either of the parents, but there was between-trait difference regarding which parent *C. flexuosa* resembled more. The peak flowering time was closer to that of *C. amara*, while the duration of flowering and the size of flowers and petals were closer to those of *C. hirsuta* (Figs. 1, 2). This trait-dependent divergence of the allopolyploid from the parental species is in accordance with the findings obtained by a study of *Nicotiana* in the laboratory (McCarthy et al. 2016), in which multiple species collected worldwide were grown in a uniform condition. Therefore, the reproductive traits of an allopolyploid and its diploid parents can diverge in both controlled and natural conditions. No environment was controlled in the present study; however, because it was conducted at a local scale in the same climatic region, the evolutionary significance of the divergence observed between the allopolyploid and its diploid parents is likely attributed to the difference in the mating system, genetics, natural selection, or microhabitat rather than to environmental differences between sites. In the following sections, we discuss the evolutionary and ecological implications of variations in the traits studied.

We recorded the flowering phenology of an allopolyploid in two consecutive years in natural habitats. *Cardamine flexuosa* consistently flowered at an intermediate time, showing a similar trend to the flowering of allopolyploids observed under controlled conditions (Anssour et al. 2009; Davis 1943; Schranz and Osborn 2004). In both years, *C. hirsuta* was the first to flower, followed by *C. flexuosa* and *C. amara*. This is similar to previous findings from *Mimulus* and *Clarkia*, in which self-fertilizing species flowered earlier compared with the outcrossing counterparts (Martin and Willis 2007; Mazer et al. 2004). Although this result was not statistically significant, the peak of flowering was narrower in the outcrossing species *C. amara* than it was in the other two selfing species in the present study. This trend persisted when the species were resolved into sites (Fig. S4) and corresponded to the known difference between outcrossing and selfing species; that is, while pollinators impose natural selection on synchronized flowering in outcrossing species (Forsyth 2003), selfing species are expected to be free from such natural selection. The large overlap between the flowering period of *C. flexuosa* and those of the parents (Fig. 1) should not hinder the reproductive isolation of *C. flexuosa*, given that it is self-fertilizing. Consistent with this interpretation, no triploids were observed in the cohabited study sites (see “Materials and methods”).

In addition to the mating system and genetic differences between species, the fine-scale environment may have affected the flowering phenology of the studied individuals. Early flowering is often associated with dry, bright, and nutrient-rich environments (Martin and Willis 2007; Mazer et al. 2004; Nord and Lynch 2009; Stanton et al. 2000; but see Callahan and Pigliucci 2002 for early onset of reproduction in shade). In fact, the habitat in the study area of the early-flowering parent *C. hirsuta* was characterized by the presence of less water, more nitrogen, and more light, while the opposite was observed for the late-flowering parent *C. amara*; the environmental condition of the *C. flexuosa* habitat was intermediate (Akiyama et al. 2019). Thus, the allopolyploid *C. flexuosa* was intermediate in both flowering phenology and habitat at the fine geographical scale.

The size of the floral organs of *C. flexuosa* was closer to that of the self-fertilizing parent *C. hirsuta* than to that of the outcrossing parent *C. amara* (Fig. 2). This result corresponds to the evolution of the selfing syndrome; selfing species have smaller flowers than the outcrossing counterparts, as a consequence of natural selection on floral morphology (Foxe et al. 2009; Shimizu and Tsuchimatsu 2015; Sicard and Lenhard 2011; Tedder et al. 2015a). Conversely, the findings of previous studies of recently emerged allopolyploids in natural habitats suggest that inheritance and/or allopolyploidization influence floral organ size. The floral organs of *Mimulus peregrinus* were larger than those of the parental diploids (Vallejo-Marín 2012). The discrepancy between this result and findings of the present study may be attributed to the time since allopolyploidization. Having emerged 10^5^–10^6^ years ago, *C. flexuosa* may exhibit a number of generations that is sufficient for the manifestation of the selfing syndrome. Whether the floral organ size of self-fertilizing allopolyploids is negatively correlated with time since allopolyploidization can be examined by a comparative field study of multiple taxa featuring different mating systems, different time since allopolyploidization, and different locations (laboratory and natural habitat). Such a study should provide a perspective on the interpretation of previous studies performed in the field or laboratory that reported that the floral organs of allopolyploids are larger than, not different from, or smaller than those of the parents (Abbott and Lowe 2004; Alexander-Webber et al. 2016; Allen 2001; Anssour et al. 2009; Benedict et al. 2012; McCarthy et al. 2016; Perný et al. 2005; Vallejo-Marín 2012; Table 2 in Vamosi et al. 2007).

In summary, the present study provides empirical quantitative data on the reproductive traits of a wild allopolyploid and its parental species in natural habitats. At the local scale, we examined the variation in floral morphology and flowering phenology in *Cardamine*. The allopolyploid exhibited a trait-dependent resemblance to the parental species. We also discussed a scenario in which the small floral organs of the self-fertilizing allopolyploid *C. flexuosa* are a product of the selfing syndrome. Such an evolutionary scenario, as well as the underlying molecular mechanisms, can be examined effectively in *Cardamine*. The genus has the advantages of consisting of different ploidy levels, of having a known time since polyploidization and mating systems for multiple species, and of the availability of rich genomic and genetic resources. Our understanding of trait variation in allopolyploids may be advanced by considering not only the inheritance and allopolyploidization, but also the mating system of these species.

## Electronic supplementary material

Below is the link to the electronic supplementary material.

Supplementary file1 (PDF 1262 kb)
