# Supplementary material for: Trait-dependent resemblance of the flowering phenology and floral morphology of the allopolyploid Cardamine flexuosa to those of the parental diploids in natural habitats
Source: J Plant Res. 2020 Jan 10;133(2):147–55. doi: 10.1007/s10265-019-01164-0 (PMC7026219; doi:10.1007/s10265-019-01164-0)
Supplement: Supplementary file 1 — Supplementary file1 (PDF 1262 kb) [file 10265_2019_1164_MOESM1_ESM.pdf]

Electronic supplementary materials

TITLE:

Trait-dependent resemblance of the flowering phenology and floral morphology of the allopolyploid *Cardamine flexuosa* to those of the parental diploids in natural habitats

JOURNAL:

Journal of Plant Research

AUTHORS:

Reiko Akiyama, Stefan Milosavljevic, Matthias Leutenegger, Rie Shimizu-Inatsugi

CORRESPONDING AUTHOR:

Rie Shimizu-Inatsugi

Department of Evolutionary Biology and Environmental Studies, University of Zurich, Winterthurerstrasse 190, CH-8057, Zurich, Switzerland

Tel.: +41 (0)44 63 54760

FAX: +41 (0)44 63 56821

Email: rie.inatsugi@ieu.uzh.ch

CONTENTS:

Figures S1-S6

Table S1

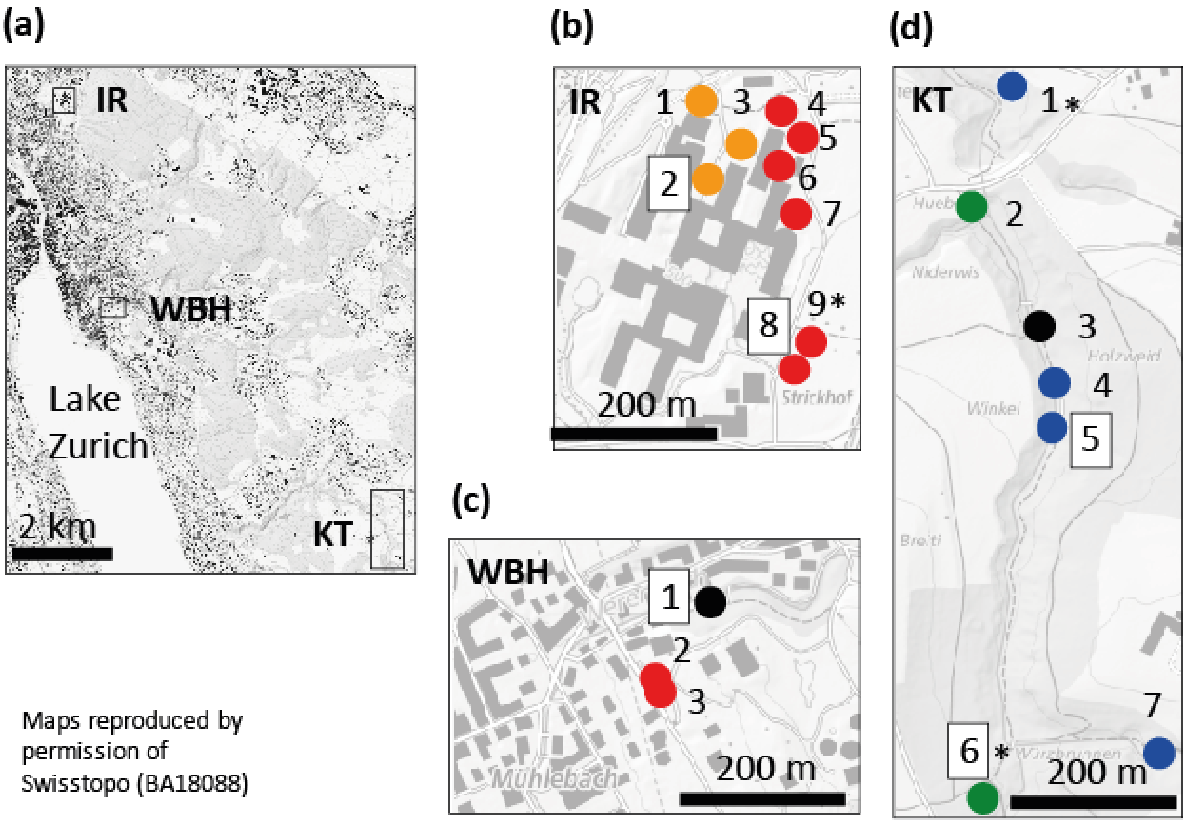

Figure S1 Locations and compositions of the study species in *Cardamine*. (a) Locations of the study areas Irchel (IR), Wehrenbach (WBH), and Küsnacht-Tobel (KT) in and around Zurich, Switzerland. (b) Locations of the study sites at IR. (c) Locations of the study sites at WBH. (d) Locations of the study sites at KT. Different colours of the circles indicate different species composition: red, *C. hirsuta* only; orange, *C. hirsuta* and *C. flexuosa*; green, *C. flexuosa* only; blue, *C. flexuosa* and *C. amara*; and black, *C. amara* only. The sites with numbers with square background correspond to where floral morphology study was conducted. The asterisks indicate the sites where flowering phenology was recorded for one of two years (see Table S1 for details)

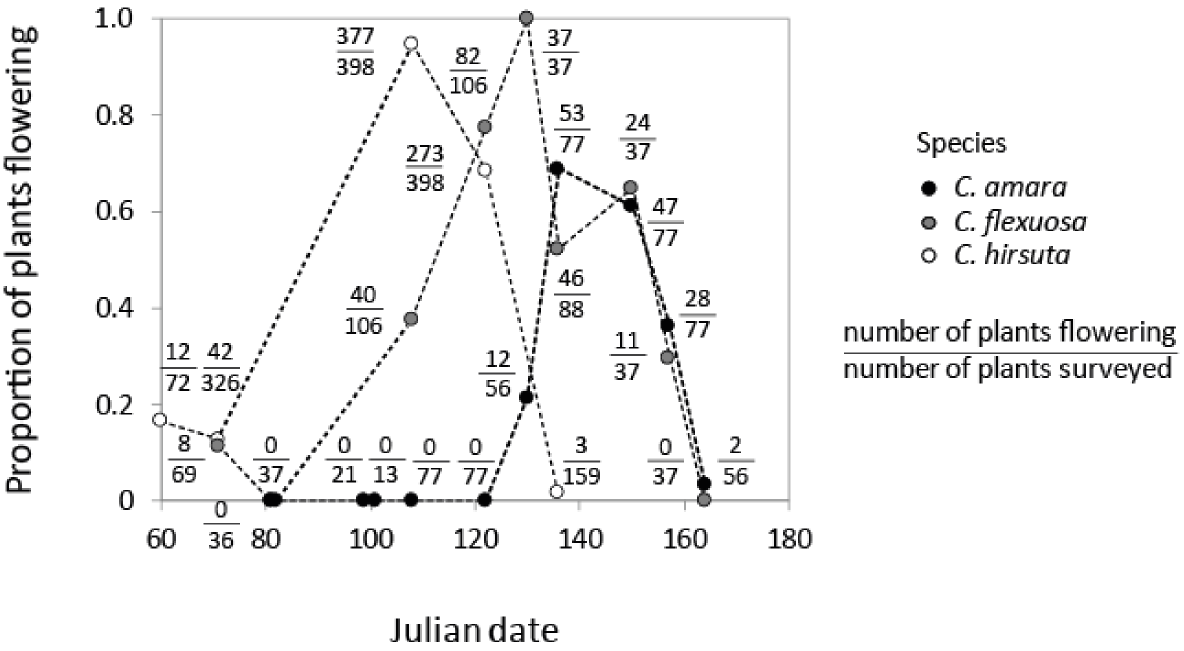

Figure S2 Proportion of flowering individuals of *Cardamine amara*, *C. flexuosa*, and *C. hirsuta* in all study sites in Switzerland in 2013. The total number of plants scored at each census ranged from 13 to 77 for *C. amara*, 37 to 106 for *C. flexuosa*, and 72 to 398 for *C. hirsuta*

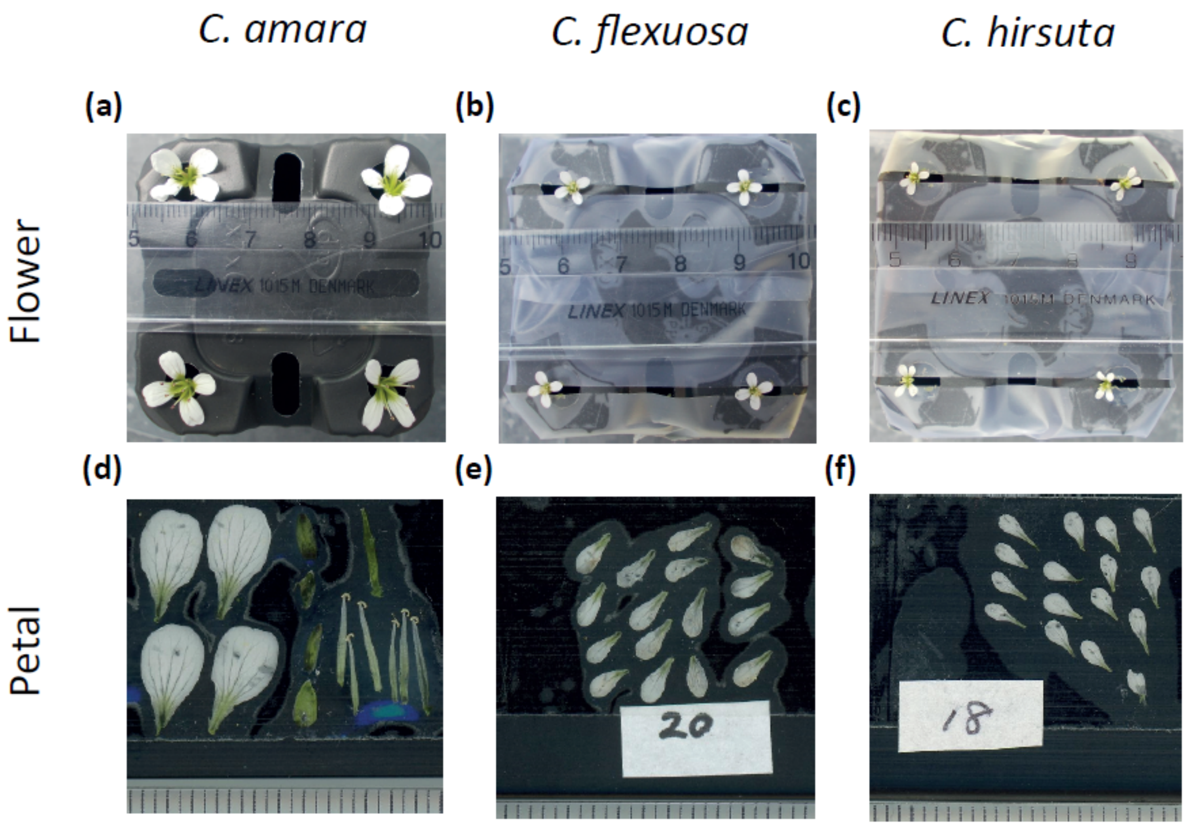

Figure S3 Photographs of flowers and scans of petals of *Cardamine amara*, *C. flexuosa*, and *C. hirsuta*. (a) flowers of *C. amara* (b) flowers of *C. flexuosa* (c) flowers of *C. hirsuta* (d) petals of *C. amara* (e) petals of *C. flexuosa* (f) petals of *C. hirsuta*

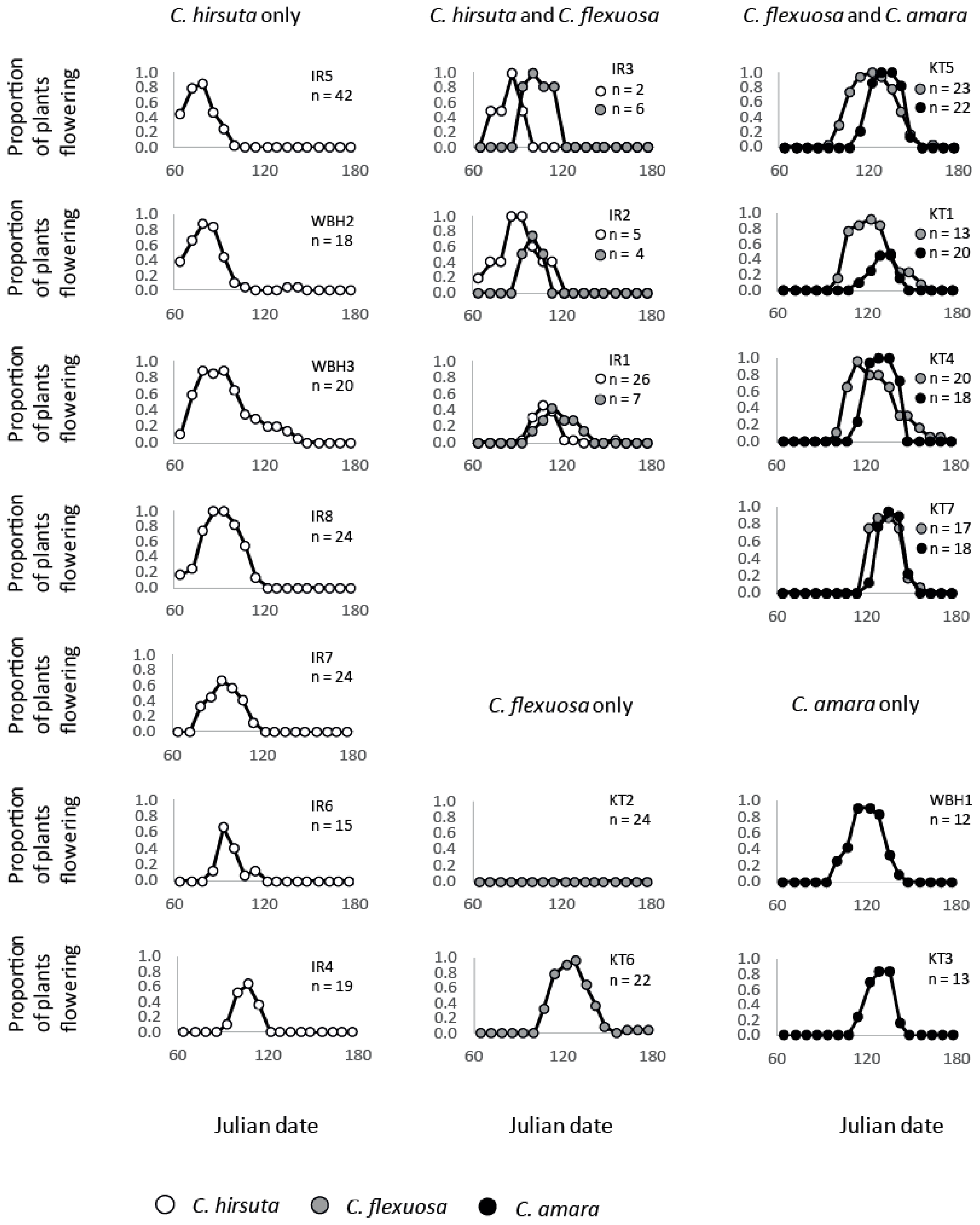

Figure S4 Proportion of flowering individuals of *Cardamine amara* (black circle), *C. flexuosa* (gray circle), and *C. hirsuta* (white circle) at each study site in Switzerland in 2013. IR = Irchel; WBH = Wehrenbach; KT = Küsnacht-Tobel

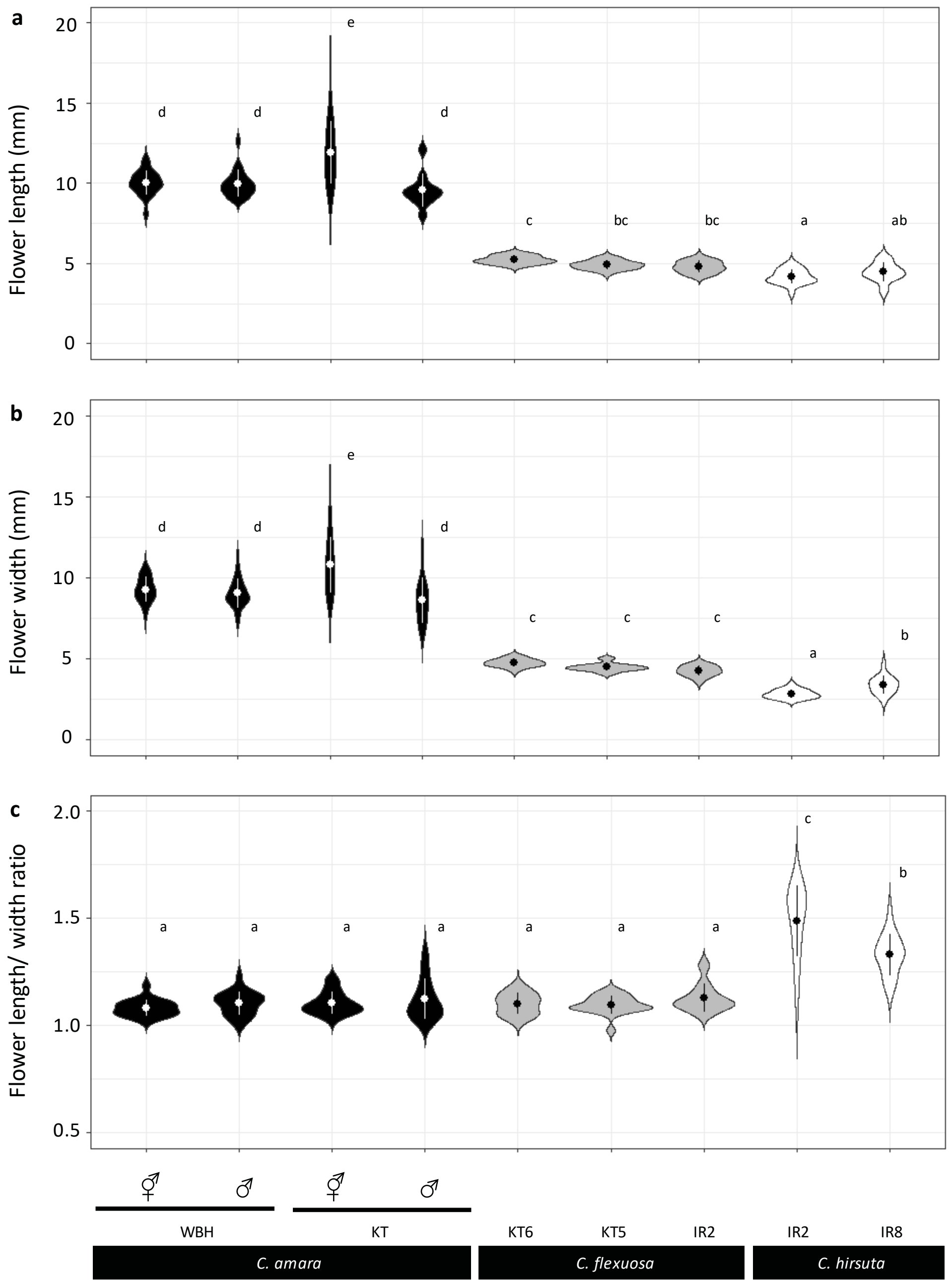

Figure S5 Dimension and length/ width ratio of flower of *Cardamine amara* (hermaphrodite and male), *C. flexuosa*, and *C. hirsuta* at each study site. IR = Irchel; WBH = Wehrenbach; KT = Küsnacht-Tobel. (a) flower length (b) flower width (c) length/ width ratio of the flower. For (a)-(c), dots and vertical lines within violin plots indicate the mean and SD and the lowercase letters in each figure indicate statistical difference based on Tukey's HSD test. The number of individuals: *C. amara*, hermaphrodite at WBH, *N* = 19; *C. amara*, male at WBH, *N* = 21; *C. amara*, hermaphrodite at KT, *N* = 19; *C. amara*, male at KT, *N* = 11; *C. flexuosa*, KT6, *N* = 15; *C. flexuosa*, KT5, *N* = 20; *C. flexuosa*, IR2, *N* = 20; *C. hirsuta*, IR2, *N* = 20; *C. hirsuta*, IR8, *N* = 18. IR = Irchel; WBH = Wehrenbach; KT = Küsnacht-Tobel

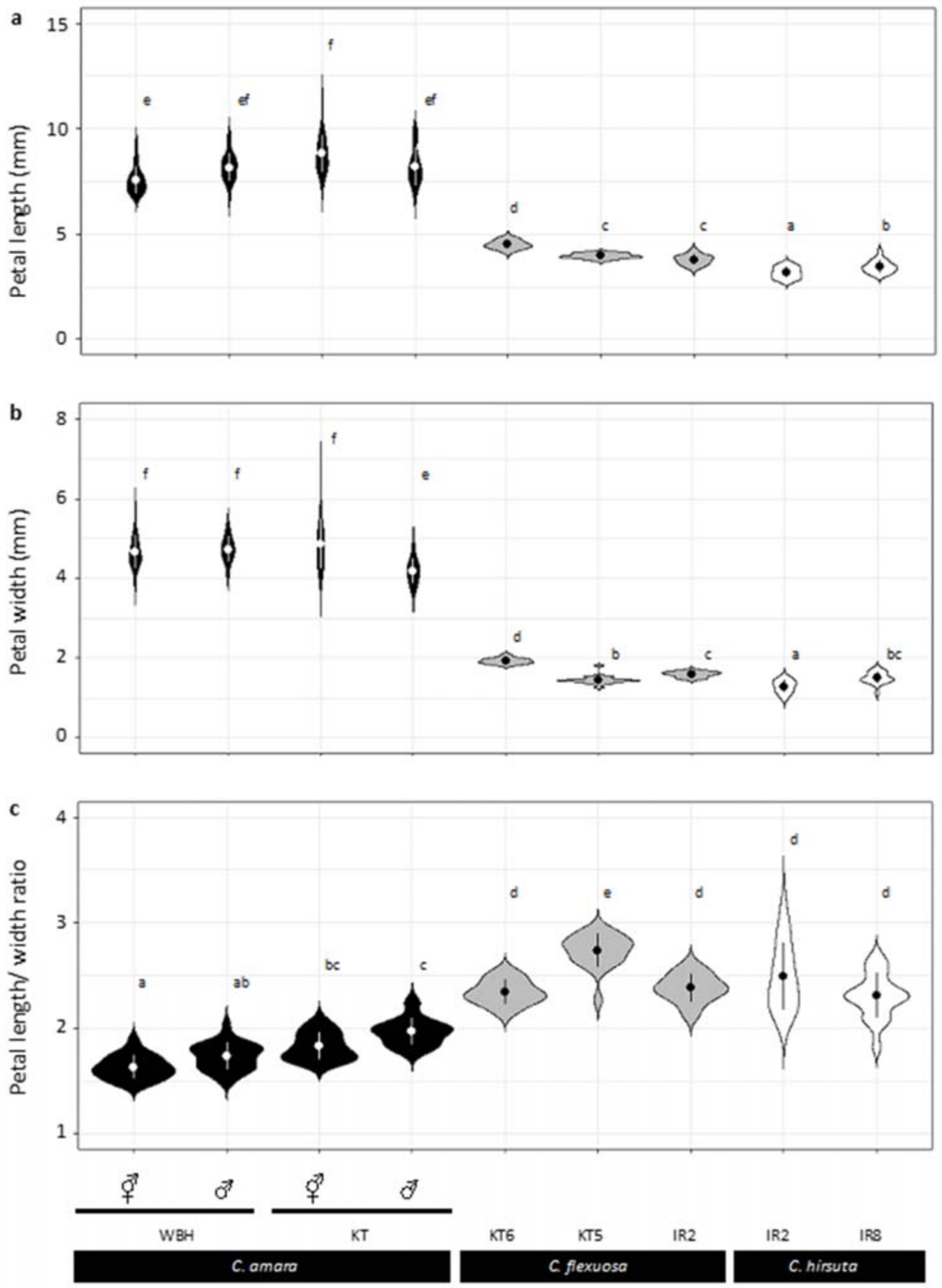

Figure S6 Dimension and length/ width ratio of petal of *Cardamine amara* (hermaphrodite and male), *C. flexuosa*, and *C. hirsuta* at each study site. IR = Irchel; WBH = Wehrenbach; KT = Küsnacht-Tobel. (a) petal length (b) petal width (c) length/ width ratio of the petal. For (a)-(c), dots and vertical lines within violin plots indicate the mean and SD and the lowercase letters in each figure indicate statistical difference based on Tukey's HSD test. The number of individuals: *C. amara*, hermaphrodite at WBH, *N* = 19; *C. amara*, male at WBH, *N* = 21; *C. amara*, hermaphrodite at KT, *N* = 20; *C. amara*, male at KT, *N* = 11; *C. flexuosa*, KT6, *N* = 15; *C. flexuosa*, KT5, *N* = 20; *C. flexuosa*, IR2, *N* = 20; *C. hirsuta*, IR2, *N* = 20; *C. hirsuta*, IR8, *N* = 20. IR = Irchel; WBH = Wehrenbach; KT = Küsnacht-Tobel

Table S1 The name of the study areas, the name of the study sites, and the number of individuals of the study species in *Cardamine* in 2013 and 2014. The number of individuals was recorded during March 4 to April 11 in 2013 and on April 10 in 2014.

		2013							2014							2016
		C. amara		*C. flexuosa*		*C. hirsuta*		FP	*C. amara*		*C. flexuosa*		*C. hirsuta*		FP	FM
Area	Site	NPO	NPA	NPO	NPA	NPO	NPA		NPO	NPA	NPO	NPA	NPO	NPA		
Irchel	IR1	0	-	25	23-25	160	1-38	hf	0	-	8	7	52	26	hf	
	IR2	0	-	6824	24-27	61	38-51	hf	0	-	332	4	64	5	hf	hf
	IR3	0	-	20	3- 6	20	18	hf	0	-	11	6	6	2	hf	
	IR4	0	-	0	-	162	34-38	h	0	-	0	-	123	19	h	
	IR5	0	-	16	1-12	189	34	hf	0	-	0	-	411	42	h	
	IR6	0	-	0	-	170	1-33	h	0	-	0	-	146	15	h	
	IR7	0	-	0	-	280	1-34	h	0	-	0	-	64	24	h	h
	IR8	0	-	0	-	46	28-40	h	0	-	0	-	425	24	h	
	IR9	0	-	0	-	276	27-40	h	NA	-	NA	-	NA	-	NA	
Wehrenbach	WBH1	750	21	0	-	0	-	a	700	12	2	-	0	-	a	a
	WBH2	0	-	0	-	132	35	h	0	-	0	-	28	18	h	
	WBH3	0	-	0	-	205	37	h	0	-	1	-	161	20	h	
Küsnacht-	KT1	223	18	0	-	0	-	a	1190	20	25	13	0	-	fa	
Tobel	KT2	0	-	211	31	0	-	f	0	-	126	24	0	-	f	
	KT3	430	13	1	-	0	-	a	800	13	1	-	0	-	a	
	KT4	247	10	60	-	0	-	fa	640	18	28	20	0	-	fa	
	KT5	210	8	61	6	0	-	fa	305	22	74	23	0	-	fa	fa
	KT6	NA	-	NA	-	NA	-	NA	0	-	169	22	0	-	f	f
	KT7	276	7	10	-	0	-	fa	640	18	39	17	0	-	fa	
	Total	2136		7228		1701			4275	103	816	136	1480	195		

IR = Irchel; WBH = Wehrenbach; KT = Küsnacht-Tobel; NA = data not available; FP = for which species (a = *Cardamine amara*, f = *C. flexuosa*, h = *C. hirsuta*) flowering phenology was recorded at each site; FM = the sites and species surveyed for floral morphology in 2016; NPO = number of plants occurred; NPA = number of plants analyzed
